# Professor Frank Abbott

**Published:** 1897-05

**Authors:** 


### PROFESSOR FRANK ABBOTT.

THE painful announcement of the sudden death of Dr. Abbott was received as this number was being prepared for the press. We are not in possession of any particulars, and are, necessarily, obliged to reserve comment until our next issue. The death of this prominent worker in dentistry will create a profound impression and leave a vacancy in dental ranks difficult to fill. Whatever criticisms may have been passed upon his work, it must be recognized that his positive character left a marked impress upon his profession.

